# A survey of the *Porrhoclubiona* Lohmander, 1944 from Central Asia (Araneae, Clubiondae)

**DOI:** 10.3897/zookeys.802.30236

**Published:** 2018-12-04

**Authors:** Yuri M. Marusik, Mikhail M. Omelko

**Affiliations:** 1 Institute for Biological Problems of the North RAS, Portovaya Str. 18, Magadan, Russia Institute for Biological Problems of the North RAS Magadan Russia; 2 Department of Zoology & Entomology, University of the Free State, Bloemfontein 9300, South Africa University of the Free State Bloemfontein South Africa; 3 Zoological Museum, Biodiversity Unit, FI-20014 University of Turku, Finland University of Turku Turku Finland; 4 Far Eastern Federal University, Sukhanova 8, Vladivostok 690950, Russia Far Eastern Federal University Vladivostok Russia; 5 Federal Scientific Center of the East Asia Terrestrial Biodiversity FEB RAS, Vladivostok, 690022 Russia Federal Scientific Center of the East Asia Terrestrial Biodiversity Vladivostok Russia

**Keywords:** *
Aranei
*, *
Clubiona
*, India, Iran, new combination, new species, new status, redescription, Tajikistan, Xinjiang

## Abstract

*Clubiona* Latreille, 1804, with more than 500 named species, is one of the largest genera of Araneae. The genus has 15 synonyms, most of which are not listed in the World Spider Catalog (2018) and unknown to many arachnologists. The most comprehensive survey of *Clubiona* sensu lato by Wunderlich (2011) also lacked a few synonyms. In this paper all genus group names described in *Clubiona* are listed with their type species. Most of these names correspond to the species groups recognised in *Clubiona* sensu lato. We agree that *Porrhoclubiona* Lohmander, 1944 (= *Clubionagenevensis*-group) deserves a status of a separate genus and provide the diagnosis of this taxon. Three species of *Porrhoclubiona* that occur in Central Asia are surveyed, and two of them are described as new to science: *P.laudata* (O. Pickard-Cambridge, 1885), **comb. n**. (♂♀, Xinjiang, Tibet, China), *P.bosmansi***sp. n.** (♂♀, Tajikistan), and *P.moradmandi***sp. n.** (♂♀, Fars, Iran). It seems that all records of *P.genevensis* L. Koch, 1866 from China refer to *P.laudata*. The records of *Clubionavegeta* Simon, 1918 from Tajikistan and Iran refer to *P.bosmansi* sp. n. and *P.moradmandi* sp. n., respectively. The following new combinations have been established: *Porrhoclubionadecora* (Blackwall, 1859), **comb. n.**, *P.diniensis* (Simon, 1878), **comb. n.**, *P.leucaspis* (Simon, 1932), **comb. n.**, *P.minor* (Wunderlich, 1987), **comb. n.**, *P.pseudominor* (Wunderlich, 1987), **comb. n.**, *P.pteronetoides* (Deeleman-Reinhold, 2001), **comb. n.**, *P.vegeta* (Simon, 1918), **comb. n.**, *P.viridula* (Ono, 1989), **comb. n.**, and *P.wunderlichi* (Mikhailov 1992), **comb. n.** (all ex. *Clubiona*). SEM study of the structure considered earlier as scopula in *Clubiona* and *Porrhoclubiona* reveals that it is represented by several lateral rows of movable macrosetae (spines) with a locking mechanism.

## Introduction

*Clubiona* Latreille, 1804 with more than 500 species (WSC 2018) is one of the largest genera of the order Araneae. There have been several attempts to split this genus either to genera and subgenera (Lohmander 1944; Wunderlich 2011) or to species groups (Mikhailov 1990, 1992, 1995, 2003; Deeleman-Reinhold 2001). One of the most distinct groups of the genus is the *Clubionagenevensis*-group (Bosmans et al. 2017) or subgenusPorrhoclubiona Lohmander, 1944 belonging to Microclubiona Lohmander, 1944. Both subgenus and genus are currently considered in the genus *Clubiona*, although both sexes have autapomorphies lacking in other clubionids. While studying spiders described by O. Pickard-Cambridge from the Himalayas, we recognised one species of *Clubiona* belonging to the *C.genevensis*-group. While tubes with types from the Himalayas are lacking any name or geographical labels it was easy to identify these specimens as *C.laudata* due to the figures in Pickard-Cambridge (1885). Because this species is very similar to *C.genevensis*, we decided to compare it to available specimens. Comparison of this species with specimens identified as *C.genevensis* from Tajikistan and southern Iran revealed differences between them as well as with syntypes of *C.laudata*. The goals of this paper are 1) to provide the first redescription of *C.laudata*, 2) the description of two new species, 3) revalidation, re-diagnosis, and re-delimitation of *Porrhoclubiona*.

## Materials and methods

Specimens were photographed with a Canon EOS 7D camera attached to an Olympus SZX16 stereomicroscope and with a SEM JEOL JSM-5200 scanning microscope at the Zoological Museum, University of Turku, Finland, and digital images were montaged using “Zerene Stacker” image stacking software. Epigynes were cleared in a 10% KOH/water solution until soft tissues were dissolved. Photographs were taken in dishes with cotton on the bottom to hold the specimens in an appropriate position. All measurements are given in millimetres. Abbreviations used are as follows:

**Fe** femur,

**Pt** patella,

**Mt** metatarsus,

**Ti** tibia,

**Ta** tarsus,

**d** dorsal,

**p** prolateral,

**r** retrolateral,

**v** ventral.

The material examined is deposited in the following institutes:

**OUMNH** The Oxford University Museum of Natural History

**ZMMU** Zoological Museum of the Moscow State University, Russia

**ZMUT** Zoological Museum, University of Turku

**MMUE** The Manchester Museum, the University of Manchester

**ZMUI** Zoological Museum, University of Isfahan

**Comparative material**: *Porrhoclubionaleucaspis* (Simon, 1932): 2♂ (ZMUT), FRANCE, *Paris*, Jardin des Plantes, on *Platanus* trunk, 13.04.1968 (Pentti Häkkilä); 1♀ (ZMUT), FRANCE, *Corsica*, St Georgis-Cauro, in litter of deciduous forest, 25.05.1972 (P. Lehtinen). *Clubionapallidula* (Clerck, 1757): 1♂ (ZMUT), FINLAND, Nauvo, Kasaholm, 10.06.1959 (P.T. Lehtinen).

## Taxonomy

### Clubiona

Taxon classificationAnimaliaAraneaeClubionidae

Latreille, 1804

Clubiona Latreille, 1804: 134 (type Araneuspallidulus Clerck, 1757).
Hirtia Thorell, 1881: 222 (type H.hatamensis Thorell, 1891).
Atalia Thorell, 1887: 54 (type A.concinna Thorell, 1887).
Tolophus Thorell, 1891: 26 (type T.submaculatus Thorell, 1891).
Paraclubiona Lohmander, 1944: 19 (type Araneacorticalis (Walckenaer, 1802).
Microclubiona Lohmander, 1944: 20 (type C.trivialis C.L. Koch, 1834).
Porrhoclubiona Lohmander, 1944: 20 (subgenus of Microclubiona, type C.clandestina Menge, 1873 (= C.genevensis L. Koch, 1866).
Hyloclubiona Lohmander, 1944: 20 (subgenus of Microclubiona, type C.comta C.L. Koch, 1839).
Heteroclubiona Lohmander, 1944: 20 (subgenus of Clubiona, type C.terrestris Westring, 1851).
Epiclubiona Lohmander, 1944: 20 (subgenus of Clubiona, type C.neglecta O. Pickard-Cambridge, 1862, not C.similis L. Koch, 1866 as indicated by Wunderlich 2011).
Euryclubiona Lohmander, 1944: 21 (subgenus of Clubiona, type C.subsultans Thorell, 1875).
Gauroclubiona Lohmander, 1944: 21 (subgenus of Clubiona, type C.coerulescens L. Koch, 1867).
Bucliona Benoit, 1977: 68 (type Clubionadubia O. Pickard-Cambridge, 1869).
Japoniona Mikhailov, 1990: 143 (C.japonica L. Koch, 1878).
Bicluona Mikhailov, 1994: 52 (subgenus of Clubiona, Liocranumjucundum Karsch, 1879).
Marmorclubiona Wunderlich, 2011: 136 (type C.marmorata L. Koch, 1866).
Breviclubiona Wunderlich, 2011: 139 (type C.brevipes Blackwall, 1841).
Anaclubiona Ono, 2010: 4 (type C.zilla Dönitz & Strand, 1906).

#### Note.

Above we have listed all names that are currently considered synonyms of *Clubiona*. Most are missing from the WCS (2018), but almost all are listed in Wunderlich (2011) and in Mikhailov (2012). One name is lacking in all three aforementioned publications, *Hirtia*. Because many genus group names correspond to the species groups and very distinct from *Clubiona* s. str., and *Clubiona* is one of the largest genera of spiders, most of the genus group names can be considered separate genera (if they are not junior synonyms). Notably, Wunderlich (2011) suggested to resurrect all genus group names in *Clubiona*, describing several genera and three family group names, two for recent species, Microclubionini Wunderlich, 2011 and Eodotinae Wunderlich, 2011, and one for the fossil genus *Eodoter* Petrunkevitch, 1858 (Eodotinae Wunderlich, 2011). Mikhailov (2012) synonymised all genera listed above with *Clubiona*.

The *Clubionagenevensis*-group fits Lohmander’s *Porrhoclubiona* Lohmander, 1944 with the type species *C.genevensis*. Wunderlich (2011) considers *Porrhoclubiona* as separate genus in Microclubionini. Here we follow Wunderlich’s subdivision of *Clubiona* sensu lato.

#### Comments.

While trying to rediagnose *Porrhoclubiona* we noticed a peculiar modification of leg I and II in females: they have a kind of scopula (Figs 2d, 3a). A similar modification was documented for *Clubionacomta* C.L. Koch, 1839 (= *Hyloclubionac.*) by Marusik and Kunt (2010). We thought that it was a diagnostic character for two related genera, but checking *Clubionapallidula*, the generotype (Fig. 2e) and some other species revealed that this character is present in many species of *Clubiona* s. l. Locket & Millidge (1951: 125) mentioned scopulae on legs I and II present in all British *Clubiona*, that it was well developed only in females and can be reduced to a single row in a smaller species. Deeleman-Reinhold (2001) also reported the presence of scopulae in the *Clubionapteronetoides*-group without specifying in which sexes.

Light microscopy (Fig. 2d, e) indicated that the modified setae cannot be considered as scopula. They are absent ventrally on the tarsus-tibia but located ventro-laterally and additionally are adpressed and not erect. SEM microscopy reveals that that “setae” in “scopula” are movable spines and have locking mechanisms (*Lm*), at least on the metatarsi and tibiae (Fig. 3e). A locking spine mechanism is known in several unrelated groups of spiders like Oonopidae, Corinnidae, Phrurolithidae, etc. (cf. Marusik et al. 2013: figs 7–10). In that groups, the locking mechanism is present in both sexes and such as ventral paired spines only. These spines are long, and when erect act as a catching basket for prey capture. The function of such spines arranged in 3–4 rows on each side of the segment is unclear.

### Porrhoclubiona

Taxon classificationAnimaliaAraneaeClubionidae

Lohmander, 1944

Porrhoclubiona Lohmander, 1944: 20 (subgenus of Microclubiona, type C.clandestina Menge, 1873 (= C.genevensis L. Koch, 1866).
Porrhoclubiona : Prószyński and Staręga 1971: 234; Sterghiu 1985: 54 (considered as subgenus).
Porrhoclubiona : Wunderlich 2011: 140 (considered as a genus).
Clubiona genevensis -group: Bosmans et al. 2017: 2.
Clubiona pteronetoides -group: Deeleman-Reinhold 2001: 96.

#### Note.

Above we listed only two of the most recent publications dealing with this species group.

#### Diagnosis.

*Porrhoclubiona* differs from all other clubionids by having modified setae on the cymbium (Figs 4a, c, d, f, g, i, 5a–c and Bosmans et al. 2017: figs 52–79), a retrolateral basal extension of the cymbium (called here a tutaculum, *Tu*, Figs 4a, c, d, f, g, i, 5g and Bosmans et al. 2017: figs 55, 59, 63, 67), a tegular groove (*Tg*) serving as a kind of conductor for the embolus (Figs 4b, c and Bosmans et al. 2017: figs 55, 59, 63, 67), the presence (Fig. 4h) of a prolateral tibial apophysis (*Pt*) which is lacking in other genera and strongly reduced, and posteriorly located subtegulum (*St*) (vs. large prolateral subtegulum in other genera). *Porrhoclubiona* differs from *Clubiona* s. str. by the smaller size, strongly protruding male chelicera (cf. Fig. 2b and Fig. 2f), shape of endites with a deep constriction (vs. unmodified endites, Fig. 2i, j), undivided short tibial apophysis of the male palp (vs. divided); brush of long modified setae on cymbium (vs. unmodified setae), filamentous embolus (vs. short, stick-like). Females of *Porrhoclubiona* differ from these of *Clubiona* by the shape of receptacles: round sclerotised (or primary, *Sr*) and round hyaline (or secondary, *Hr*) receptacles (vs. both pairs of receptacles elongate). Females of *Porrhoclubiona* have no such distinct differences from other genera as males.

#### Comments.

Aside from those mentioned in the diagnosis, characters that separate *Porrhoclubiona* from all other genera previously considered in *Clubiona*, such as the presence of a patch/brush of modified setae on the cymbium, a cymbial extension that can be considered a tutaculum (*Tu*) and a tegular groove (*Tg*) serving as a conductor, a simple retrolateral tibial apophysis, and the presence of a prolateral apophysis, a few more characters should be mentioned. The two genera differ by spination of leg I: *Porrhoclubiona* is lacking metatarsal spines which are present in *Clubiona* and have fewer ventral tibial spines (cf. Fig. 2d and Fig. 2e). *Porrhoclubiona* has better developed “scopula”, which stretch along the entirety of tibia I, while in *Clubiona* it occupies only the distal ½ of the tibia (cf. Fig. 2d and Fig. 2e).

Although the retrolateral tibial apophysis of the male palp looks simple, from SEM figures it is rather broad (Fig. 5e) and the tip has a kind of filamentous extension (*Fl*). This tip can be long, like in *P.vegeta* (Bosmans et al. 2017: fig. 65) or *P.moradmandi* sp. n. (Fig. 5e), or rather short like in *P.bosmansi* sp. n. or *P.genevensis* (Figs 4c, 5f). Although the base of the embolus looks like one sclerite, in fact it is composed of two sclerites (Figs 4b, e, h, 9c, d, 10b’), heFl (*Ts*) and the base of the embolus (*Eb*).

Some species can be separated based on the proportions of the cymbial setae. *Porrhoclubionalecucaspis* has distinctly longer basal part of the setae (*Sb*) than *P.moradmandi* sp. n. and *P.bosmansi* sp. n. (cf. Fig. 5c and Fig. 5a, b, respectively).

The haematodocha in *Porrhoclubiona* is rather large, but the subtegulum is strongly reduced and located posterior to the embolus base (Figs 4h, 6a, d, 7a, d); however, it is not large and or located prolaterally as in all other *Clubiona* s. l. It appears that species in this genus can be separated by the shape of the sperm duct course and relative width of the sperm duct (cf. Fig. 9c and Fig. 9d).

While studying morphology of the *Porrhoclubiona* with SEM, we found several notable characters:

– The femur has few bald areas (*Ba*), not covered with a transversal furrow as other parts of the cuticle (Fig. 3d). Such bald areas are known in several unrelated families.

– The tarsal organ (*To*) in *Porrhoclubiona* is (if we recognized it correctly) slit like (Fig. 3c).

– The trichobothrial base has five transversal ridges (Fig. 3g).

– *Porrhoclubionamoradmandi* sp. n. has modified short setae (*Ms*) on the cymbium (Fig. 5d) (may also be peculiarly broken setae).

#### Composition.

Bosmans et al. (2017) listed eight species belonging to the *Clubionagenevensis*-group. We establish a new combination for all of them except the generotype *Porrhoclubionadecora* (Blackwall, 1859), comb. n. (Madeira, Azores), *P.diniensis* (Simon, 1878), comb. n. (western Mediterranean), *P.genevensis* (L. Koch, 1866) (West Palaearctic?), *P.leucaspis* (Simon, 1932), comb. n. (western North Africa, Western Europe), *P.minor* (Wunderlich, 1987), comb. n. (the Canaries), *P.pseudominor* (Wunderlich, 1987), comb. n. (the Canaries), *P.vegeta* (Simon, 1918), comb. n. (Mediterranean or West Palaearctic) and *P.wunderlichi* (Mikhailov, 1992), comb. n. (Mongolia). Two species assigned to this group by Wunderlich (2011) were overlooked by Bosmans et al. (2017): *P.pteronetoides* (Deeleman-Reinhold, 2001), comb. n. and *P.viridula* (Ono, 1989), comb. n. both from SE Asia. Deeleman-Reinhold (2001) considered these two species in a separate group, the *Clubionapteronetoides*-group. Males of *P.pteronetoides* and *P.viridula* have dorsal abdominal scuta unknown in other species of the group, and possibly lack modified setae on the cymbium.

Aside from the species mentioned above, we consider three more species in this genus, *P.laudata* (O. Pickard-Cambridge, 1885), comb. n., ex. *Clubiona* and two new species, *P.bosmansi* sp. n. and *P.moradmandi* sp. n.

### Porrhoclubiona laudata

Taxon classificationAnimaliaAraneaeClubionidae

(O. Pickard-Cambridge 1885) comb. n.

**Figure 1.**
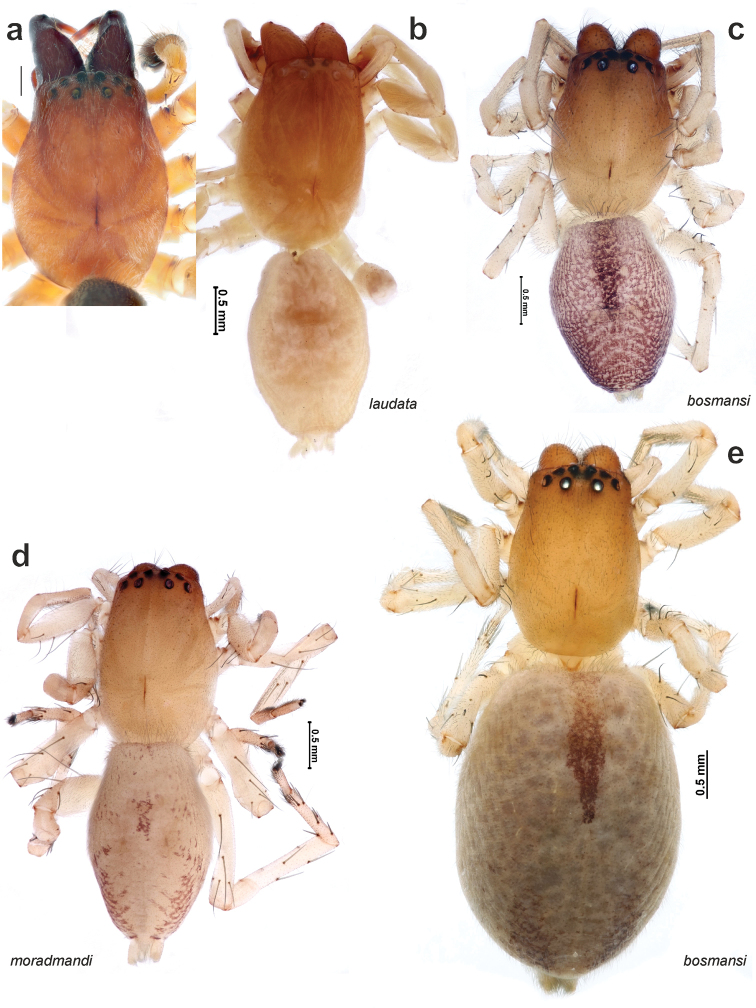
General appearance of *Clubionapallidula* (**a**), *Porrhoclubionalaudata* (**b**), *P.bosmansi* sp. n. (**c, e**) and *P.moradmandi* sp. n. (**d**). **a** prosoma, dorsal **b–d** male habitus, dorsal **e** female habitus, dorsal.

Figs 1b , 6a–c , 10c

Clubiona laudata O. Pickard-Cambridge, 1885: 23, pl. 2, f. 16 (♂♀).
Clubiona genevensis : Zhou et al. 1983: 157, f. 8a–d (♂♀); Hu and Wu 1989: 305, f. 244.1–4 (♂♀); Zhang and Hu 1989: 58, f. 6, 21 (♂♀); Song et al. 1999: 416, f. 245R-S, 248L–M (♂♀); Hu 2001: 287, f. 166.1–4 (♂♀) (all misidentifications).

#### Material examined.

Lectotype ♂ (designated here) and paralectotype ♂ (OUMNH) with a label “B[ottle] 381, v[ial] 1”, label reads “Road from Yarkand to Bursi, May 28^th^ to June 17^th^, 1874”.

#### Note.

All species described by O. Pickard-Cambridge (1885) based on materials from the Second Yarkand Mission are lacking labels with species names or geographical localities. The WSC (2018) indicates that the species was described based on the male only, although Pickard-Cambridge (1885: 24) also described a female. The species distribution is indicated as China (Yarkand), although Pickard-Cambridge (1885) mentioned the species was described based on specimens collected on the road from Yarkand (lying in SW Xinjiang, China) to Bursi (lying in the Leh District of the Jammu and Kashmir, India).

#### Diagnosis.

*Porrhoclubionalaudata* differs from *P.leucaspis* by the conical tibial apophysis (vs. broad and rounded at the tip, Figs 4i, 6e) and thinner basal part of the embolus. Tibetan species differ from other species occurring in Central Asia by the large palp (cf. Figs 10b–d).

#### Description.

Male (lectotype). Carapace 2.11 long, 1.41 wide. Abdomen 1.98 long, 1.29 wide. Total length 4.10. Carapace brown, darker in head area, fovea dark-brown, thin.

Labium, maxillae, chelicerae, and sternum yellowish. Chelicerae with 2 promarginal teeth.

Leg lengths

**Table:** 

	Fe	Pa	Ti	Mt	Ta	Total
I	1.13	0.70	1.14	0.74	0.46	4.17
II	1.36	0.79	1.44	0.94	0.51	5.04
III	1.06	0.54	0.93	0.89	0.44	3.86
IV	1.46	0.73	1.24	1.43	0.36	5.22

Leg spination

**Table:** 

	Fe	Pa	Ti	Mt
I	3d 1p	–	1-1v	–
II	3d 1p	–	1-1v	0-1v
III	3d 1p 1r	–	1p 1r 1-0v	2d 2p 1r 2-2v
IV	3d 1p 1r	1r	2p 2r 1-0v	4p 3r 2-2v

Abdomen without distinct pattern.

Palp as in Figs 6a–c, 10c. Tibial apophysis gradually tapering, subconical; anterior margin of cymbium broad; base of embolus (*Be*) equal to ½ of tegular length, basal part of embolus (*Eb*) as long as approx. 2/3 of the base.

Female. Lacking among type series. Pickard-Cambridge (1885) described it as: “The female is rather larger, but resembles the male in colours and markings, except that the abdomen is less marked and streaked with rusty red; the form of the genital aperture, which is quite small, is characteristic”. Description of *C.genevensis* from Western China seems to refer to this species. Epigyne as long as wide.

#### Distribution.

Exact type locality is unknown and in either in southwestern Xinjiang (China) or in adjacent Northeastern Jammu and Kashmir (India). It seems that all records of *C.genevensis* from China (Xinjiang and Tibet) refer to this species.

### Porrhoclubiona bosmansi sp. n.

Taxon classificationAnimaliaAraneaeClubionidae

http://zoobank.org/99107F93-8949-4D57-AD13-061A26879084

Figs 1c, e , 2a–d, j , 3 , 4a–c , 5a, g , 7d–f , 8d–f , 9a , 10d

Clubiona vegeta : Andreeva 1976: 77.

#### Types.

Holotype ♂ and paratype ♂ (ZMMU) TAJIKISTAN, **Khalton** area, Dangara Distr, Sanglogh (= Sanglok), Mt. Range, above Shar-Shar Pass, 38°17.937'N, 69°13.598'E, 1700–2060 m, 29 Apr 2015 (Y.M. Marusik), 1♀ (ZMMU) TAJIKISTAN, **Khalton** area, environs of Khovaling, Obimazar River, 38°20.940'N, 69°58.194'E, 1413 m, gravely river shore with some bushes, 27 Apr 2015 (Y.M. Marusik).

#### Etymology.

The specific name is a patronym in honour of our friend and colleague Robert Bosmans (Gent, Belgium) who made significant contributions to the study of the *Clubionagenevensis*-group.

#### Diagnosis.

*Porrhoclubionabosmansi* sp. n. differs from *P.laudata* by having a smaller carapace (1.7–1.77 vs. 2.11), smaller palp (cf. Fig. 10c and Fig. 10d) and thinner tibial apophysis. The new species differs from *P.moradmandi* sp. n. by the smaller palps and shorter modified cymbial hairs (cf. Fig. 5a and Fig. 5b), fewer pro- and retrolateral spines on metatarsus III (2-2 vs. 3-3), and inclined anterior edge of the embolic base (vs. almost horizontal, cf. Fig. 10b’ and Fig. 10d’). Female of *P.bosmansi* sp. n. differs from those of *P.moradmandi* sp. n. by the shape of the epigynal fovea, which is more transverse and lacking posterior notch (cf. Fig. 8a–c, d–f). Females of the two species differ by the shape of the copulatory ducts and relative position of hyaline and sclerotised receptacles: sclerotised receptacles located anterior to the hyaline receptacles in *P.bosmansi* sp. n. and posteriorly in *P.moradmandi* sp. n. (cf. Fig. 9a and 9b).

#### Description.

Male (holotype-paratype). Total length 3.55–3.63. Carapace 1.71–1.77 long, 1.11–1.29 wide. Abdomen 1.86–1.88 long, 1.23–1.30 wide. Carapace light-brown, cephalic area darker. Labium, maxillae, chelicerae brown. Sternum yellowish. Chelicerae with one prolateral tooth, retrolateral teeth absent.

Leg lengths (paratype).

**Table:** 

	Fe	Pa	Ti	Mt	Ta	Total
I	1.07	0.60	1.03	0.71	0.41	3.83
II	1.17	0.59	1.01	0.83	0.36	3.96
III	1.00	0.50	0.79	0.86	0.36	3.50
IV	2.88	0.64	1.07	3.50	0.43	8.52

Leg spination

**Table:** 

	Fe	Pa	Ti	Mt
I	3d 1p	–	1-0v	1-1v
II	3d 1p	–	1-2r	1-1v
III	3d 1p 1r	–	1p 1r 1-0v	2p 2r 1-2v
IV	3d 1p 1r	1r	2p 2r 1-0v	4p 4r 2-1v

Abdomen yellow-reddish at dorsal part with dark-reddish cordial mark. Lateral sides of abdomen reddish, ventral side yellowish.

Palp as in Figs 4a–c, 5a, g, 7d–f, 10d. Tibial apophysis triangular, wider than long; anterior edge of cymbium almost flat (horizontal, not rounded); setae in cymbial brush not dense, approx. 1/3 of cymbial length; anterior part of tegulum (*At*) longer than base of embolus (*Be*); posterior edge (*Pb*) of the basal part of embolus inclined as well as anterior part of embolic base.

Female. Carapace 2.1 long, 1.43 wide. Abdomen 3.38 long, 2.4 wide. Total length 5.5. Coloration as in males, but somewhat lighter. Chelicerae with 4 prolateral and 3 retrolateral teeth.

Leg lengths

**Table:** 

	Fe	Pa	Ti	Mt	Ta	Total
I	1.07	0.57	0.89	0.71	0.43	3.67
II	1.21	0.71	1.00	0.71	0.40	4.04
III	1.14	0.57	0.64	0.81	0.41	3.58
IV	1.57	0.69	1.14	1.36	0.50	5.26

Leg spination

**Table:** 

	Fe	Pa	Ti	Mt
I	3d 1p	–	1-0v*	–*
II	3d 1p	–	1-2v*	0-1v*
III	3d 1p 1r	–	1p 1r 1-0v	3p 3r 0-1v
IV	3d 1p 1r	1r	3p 3r 1-1v	4p 4r 1-1v

Epigyne as in Figs 8d, e, 9a. Fovea oval, more than twice as wide as long, posterior notch absent; translucent sclerotised receptacles (*Sr*) spaced by approx. one radius in intact epigyne; hyaline receptacles (*Hr*) located posterior to sclerotised receptacle; hyaline receptacles 1.3 times larger than sclerotised receptacles; loop of copulatory duct (*Dl*) directed posteriorly and spaced from each other by approximately one diameter.

#### Distribution.

Hatlon Province of Tajikistan.

### Porrhoclubiona moradmandi sp. n.

Taxon classificationAnimaliaAraneaeClubionidae

http://zoobank.org/B5FF090D-1086-412B-842D-68179EACF675

Figs 1d , 4d–f , 5b, d, e , 7a–c , 8a–c , 9b, d , 10b

#### Types.

Holotype ♂ and paratype ♀ (MMUM), paratypes 2♂ (ZMUI), 14 ♂ 4juv (ZMMU), IRAN, **Fars** Prov., Shiraz City, nearby Qur’an Gate, 29°38'08"N, 52°33'42"E, leaf and pine needle litter in small park, 19 Dec 2013 (Y.M. Marusik).

#### Etymology.

The specific name is a patronym in honour of the well-known Iranian arachnologist, Professor Majid Moradmand (Isfahan).

#### Diagnosis.

The new species differs from *P.bosmansi* sp. n. by the less intense colouration of the male (cf. Figs 1c & 1d). Males of this species can be distinguished by the larger palp (cf. Figs 7a–c & 7d–f and 10b & 10d), horizontal orientation of embolic base anterior edge and posterior edge of the basal part of the embolus (vs. inclined (cf. Figs 10b’ and 10d’)) and relatively longer tibia – length/width ratio approx. 2 (vs. shorter, ratio ca. 1.5). Females of *P.moradmandi* sp. n. can be distinguished from *P.bosmansi* sp. n. by the shape of the epigyne: epigynal fovea pentagonal (vs. oval) with distinct posterior notch (*vs.* lacking), anterior position of hyaline receptacles (vs. sclerotised receptacle located anteriorly), anteriorly directed loop of copulatory duct (vs. posteriorly). *Porrhoclubionamoradmandi* sp. n. is very similar to *P.leucaspis* by the abdominal pattern, palp shape, and particularly by having a filamentous extension (*Fl*) of the tibial apophysis, although the female differs by the shape of the copulatory ducts and receptacle proportions (cf. Fig. 8a–c and Fig. 9b and Bosmans et al. 2017: figs 45–51).

#### Description.

Male. Total length 3.38–4.67. Carapace 1.70–2.17 long, 1.17-1.64 wide. Abdomen 1.57–2.50 long, 1.0–1.57 wide. Carapace yellow to light brown without pattern, Labium, maxillae and chelicerae light brown. Sternum yellow. Chelicerae with one promarginal, retromarginal teeth absent.

Leg lengths (paratype with carapace 2.17 long)

**Table:** 

	Fe	Pa	Ti	Mt	Ta	Total
I	1.27	0.79	1.29	0.86	0.50	4.70
II	1.57	0.86	1.64	1.07	0.53	5.67
III	1.21	0.64	0.93	0.97	0.43	4.18
IV	2.88	0.77	1.29	3.50	0.50	8.94

Leg spination

**Table:** 

	Fe	Pa	Ti	Mt
I	3d 1p	–	1-0v	–
II	3d 1p	–	1-2v	1-1v
III	3d 1p 1r	–	1p 1r 1-0v	3p 3r 1-1v
IV	3d 1p1r	1r	2p 2r 1-0v	4p 4r 1-1v

Abdomen yellow with greyish V-shaped stripes (indistinct due to poor condition of the specimen) dorsally.

Palp as in Figs 4d–f, 5b, d, e, 7a–c, 9d, 10b. Tibial apophysis subtriangular, wider than long; tip with filamentous extension (*Fl*); anterior edge of cymbium rounded, with one distinct macroseta; modified setae of cymbial brush dense and long almost ½ of cymbial length; basal part of embolus ca. ½ of embolus base height, anterior edge of embolic base and posterior edge of basal part of embolus horizontal; base of embolus shorter than anterior part of tegulum.

Female. Total length 4.08. Carapace 1.93 long, 1.29 wide. Abdomen 2.07 long, 1.33 wide.

Coloration as in males. Chelicerae with 3 or 4 promarginal and 4 retromarginal teeth.

Leg lengths

**Table:** 

	Fe	Pa	Ti	Mt	Ta	Total
I	0.94	0.57	0.77	0.59	0.39	3.26
II	1.03	0.60	0.93	0.64	0.43	3.63
III	0.94	0.50	0.60	0.73	0.36	3.13
IV	2.88	0.67	1.03	3.50	0.43	8.51

Leg spination

**Table:** 

	Fe	Pa	Ti	Mt
I	3d 1p	–	1-0v	–
II	3d 1p	–	2-2v	0-1v
III	3d 1p 1r	–	1p 1r 1-0v	3p 2r 0-1v
IV	3d 1p 1r	1r	3p 3r 1-1v	3p 3r 1-1v

Epigyne as in Figs 8a–c, 9b. Fovea pentagonal with deep posterior notch; translucent receptacles spaced by less than radius in intact epigyne; copulatory duct well distinct in ventral view; hyaline receptacles located anteriorly from the sclerotised receptacles; loop of copulatory duct directed anteriorly; mesal part of copulatory ducts spaced by more than 3 times their diameters.

#### Distribution.

It is known from the type locality only.

**Figure 2.**
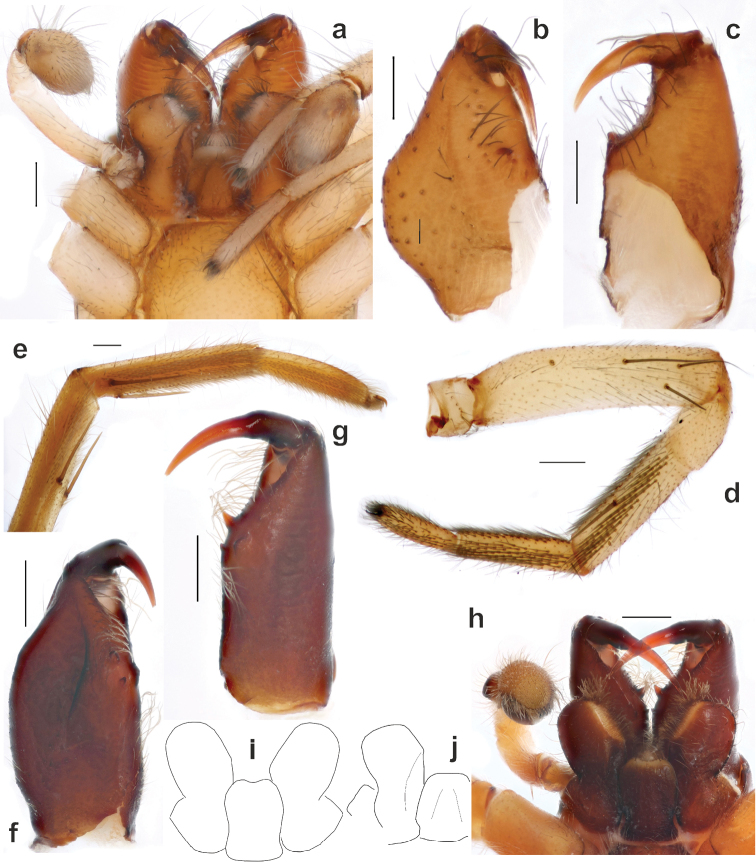
Somatic characters of *Porrhoclubionabosmansi* sp. n. (**a–d, j**) and *Clubionapallidula* (**e, f–i**). **a, h** anterior part of male prosoma, ventral, showing mouth parts **b, f** left male chelicera, mesal **c, g** left male chelicera, posterior **e** female tibia–tarsus I, prolateral **d** female leg I, prolateral **i–j** maxilla and labium, ventral.

**Figure 3.**
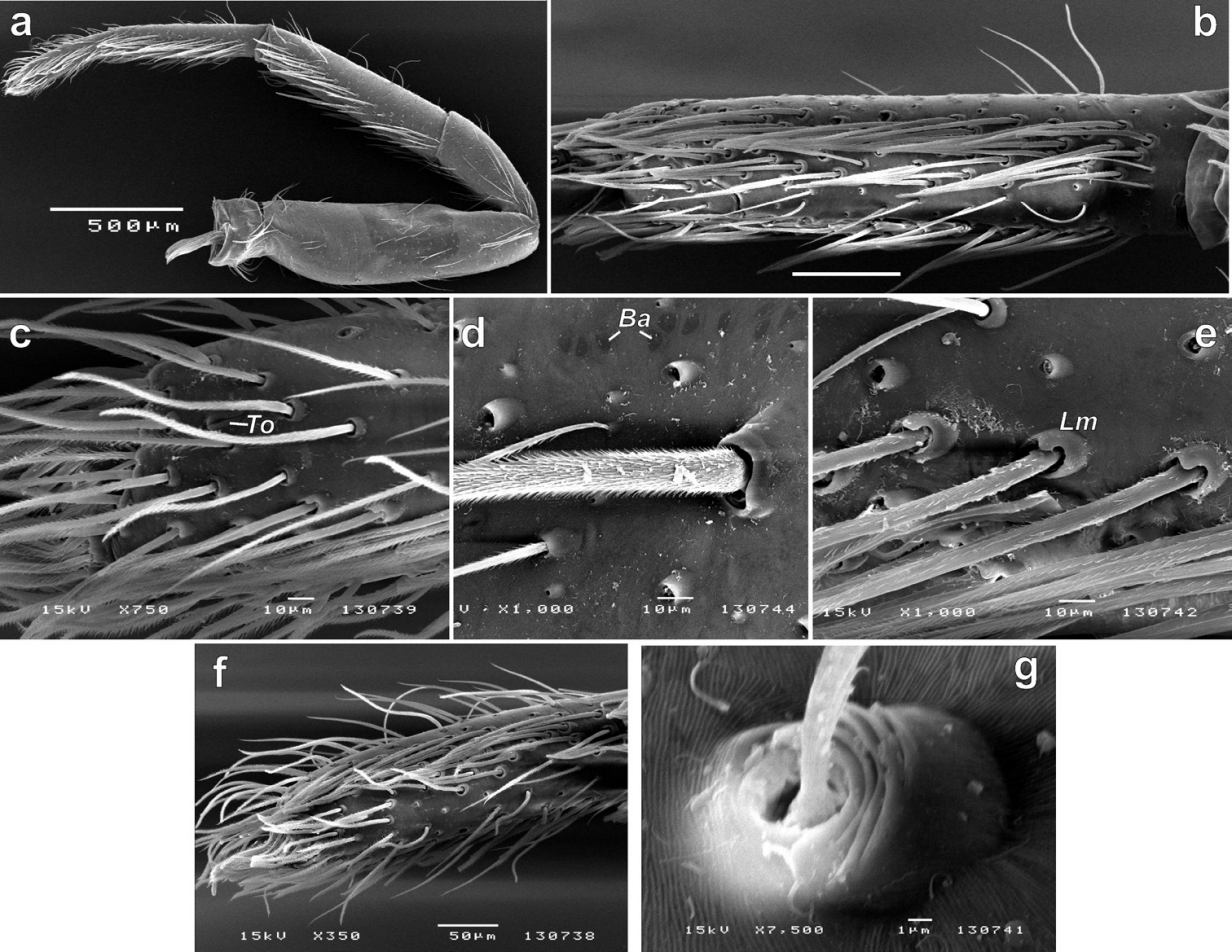
SEM micrographs of the female leg I of *Porrhoclubionabosmansi* sp. n. **a** whole leg, prolateral **b** metatarsus, subventral **c** distal part of tarsus, lateral, showing tarsal organ **d** femur, showing bold areas and spine **e** tibia, showing spines with locking mechanism **f** tarsus, subventral, showing spines lacking locking mechanism **g** trichobothrium. Abbreviations: *Ba*–bald areas, *Lm*–locking mechanism, *To*–tarsal organ.

**Figure 4.**
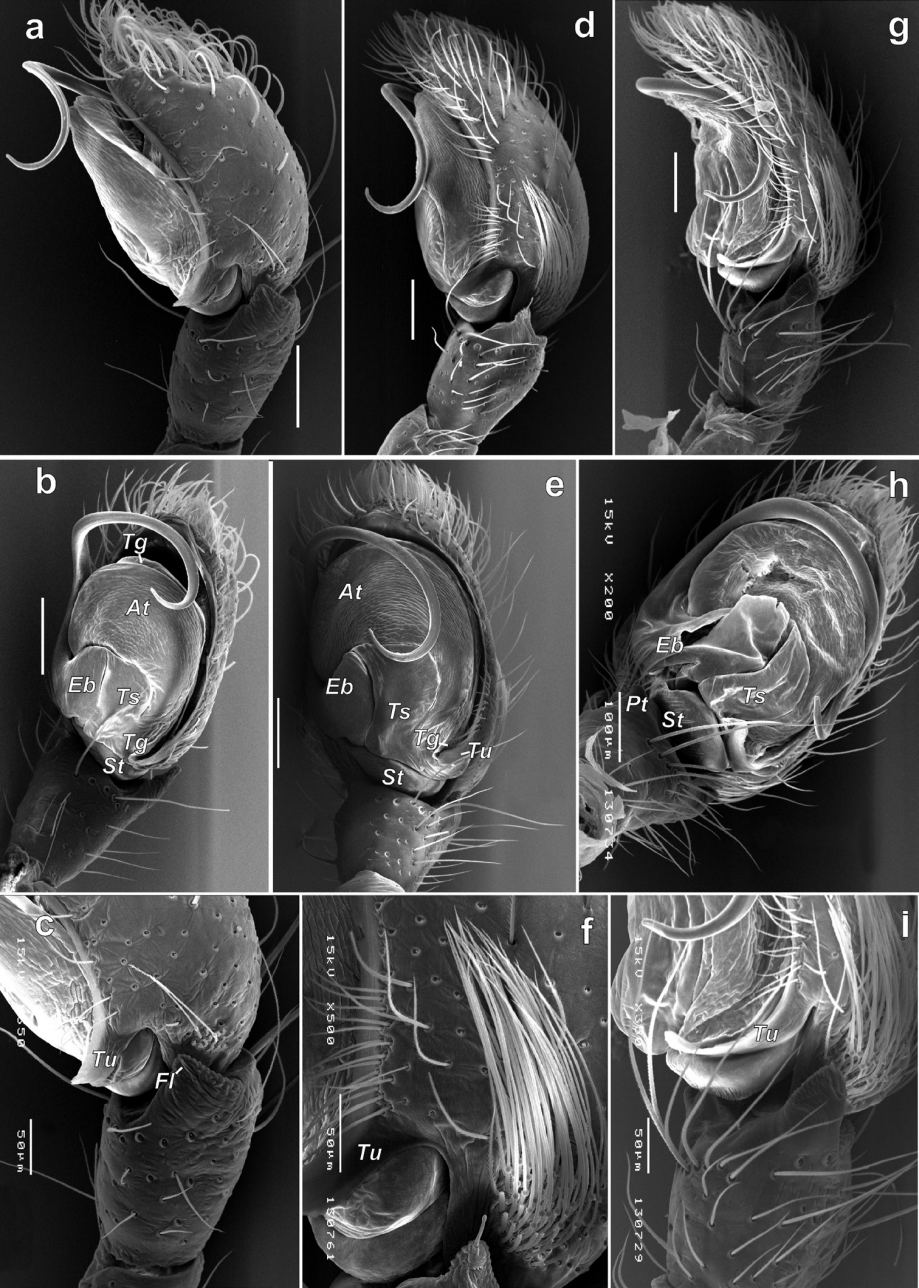
SEM micrographs of the male palp of *Porrhoclubionabosmansi* sp. n. (**a–c**), *P.moradmandi* sp. n. (**d–f**) and *P.leucaspis* (**g–i**). **a, d, g** retrolateral **b, e** retro-ventral h ventral **c, i** tibia and proximal part of bulb and cymbium; retrolateral **f** cymbium and part of tegulum, retrolateral. Abbreviations: *At*–anterior part of tegulum, *Eb*–base of embolus, *Fl*–filamentous extension, *Pt*–prolateral tibial apophysis, *St*–subtegulum, *Tg*–tegular groove, *Ts*–sclerotised part of tegulum, *Tu*–tutaculum.

**Figure 5.**
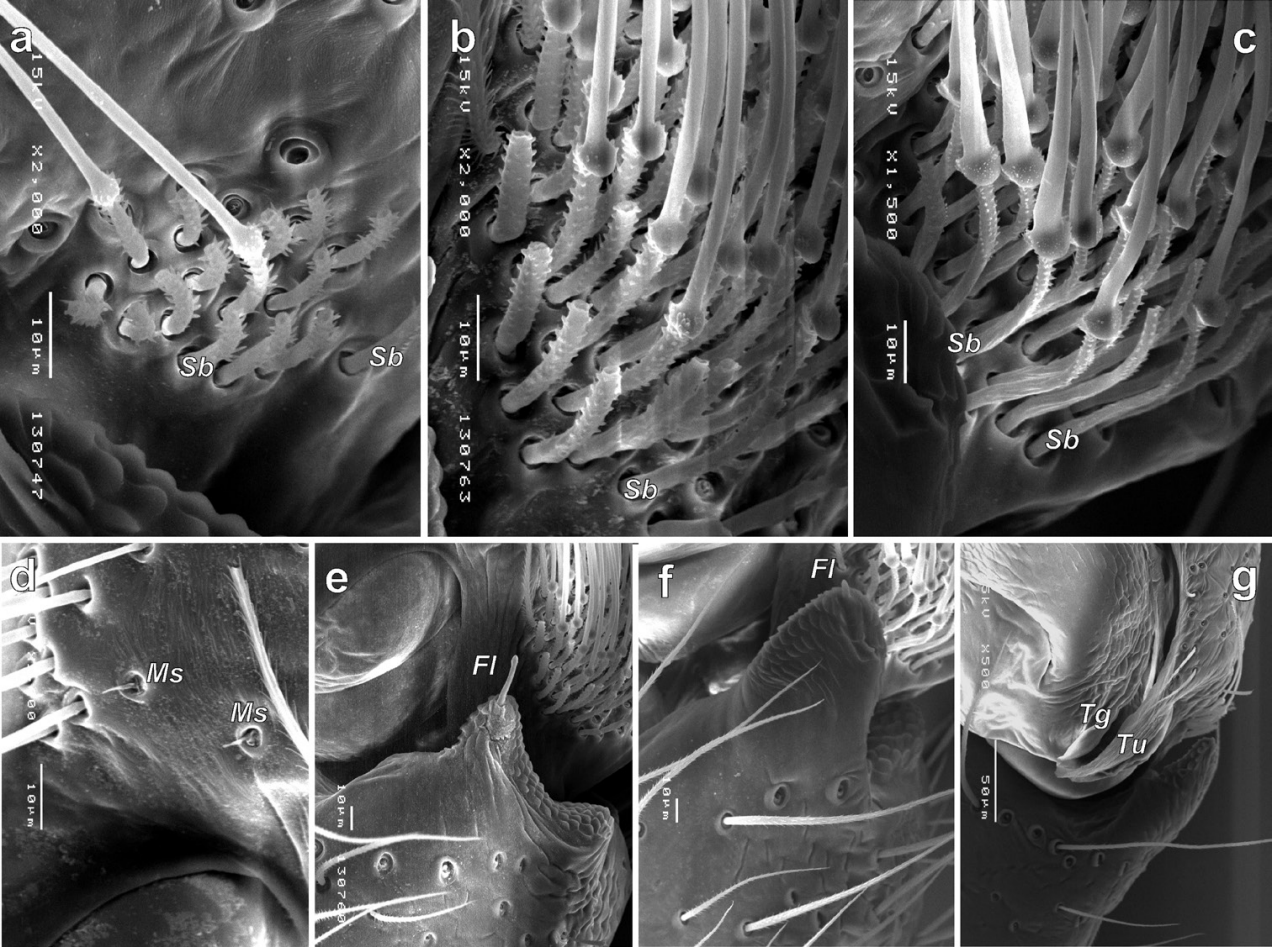
SEM micrographs of the male palp of *Porrhoclubionabosmansi* sp. n. (**a, g**), *P.moradmandi* sp. n. (**b, d, e**) and *P.leucaspis* (**c, f**). **a–c** modified cymbial setae **d** proximal part of cymbium with modified short setae **e–f** tibial apophysis **g** basoretrolateral part of cymbium showing tutaculum and tegular groove. Abbreviations: *Fl*–filamentous extension, *Ms*–modified short setae, *Sb*–basal part of setae, *Tg*–tegular groove, *Tu*–tutaculum.

**Figure 6.**
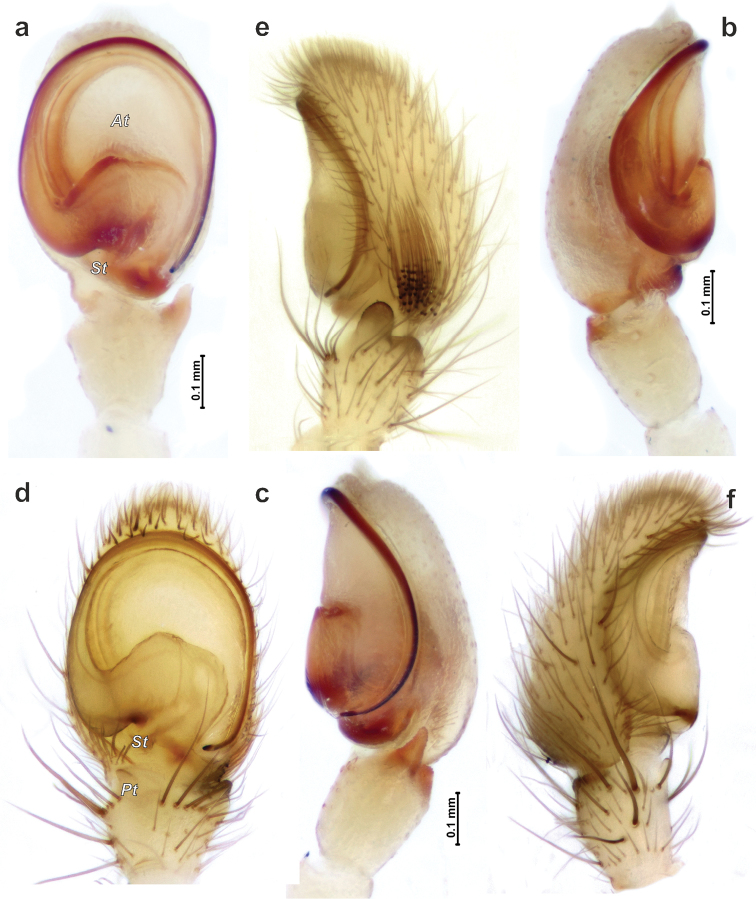
Male palp of *Porrhoclubionalaudata* (**a–c**) and *P.leucaspis* (**d–f**). **a, d** ventral **b, f** prolateral **c, e** retrolateral. Abbreviations: *At*–anterior part of tegulum, *Pt*–prolateral tibial apophysis, *St*–subtegulum.

**Figure 7.**
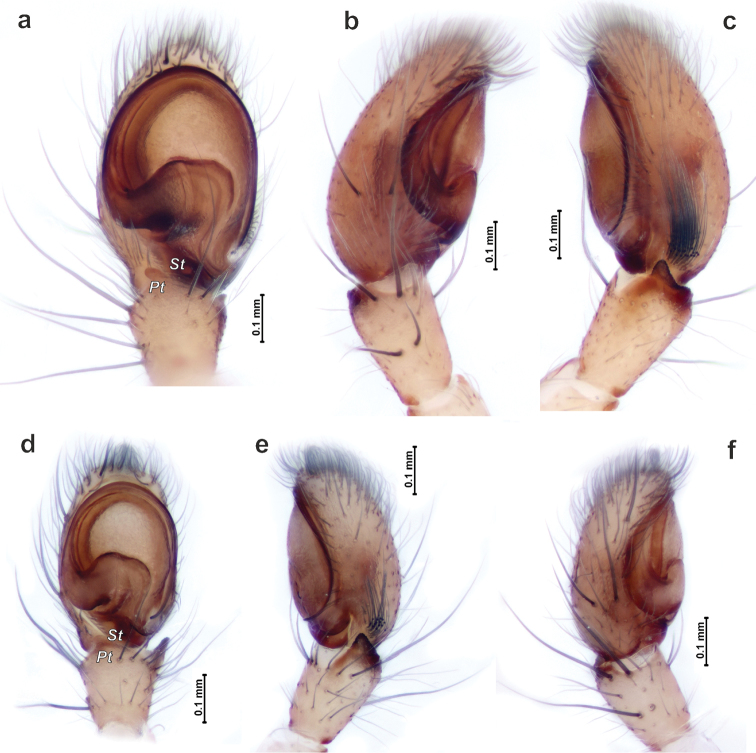
Male palp of *Porrhoclubionamoradmandi* sp. n. (**a–c**) and *P.bosmansi* sp. n. (**d–f**). Abbreviations: *Pt*–prolateral tibial apophysis, *St*–subtegulum.

**Figure 8.**
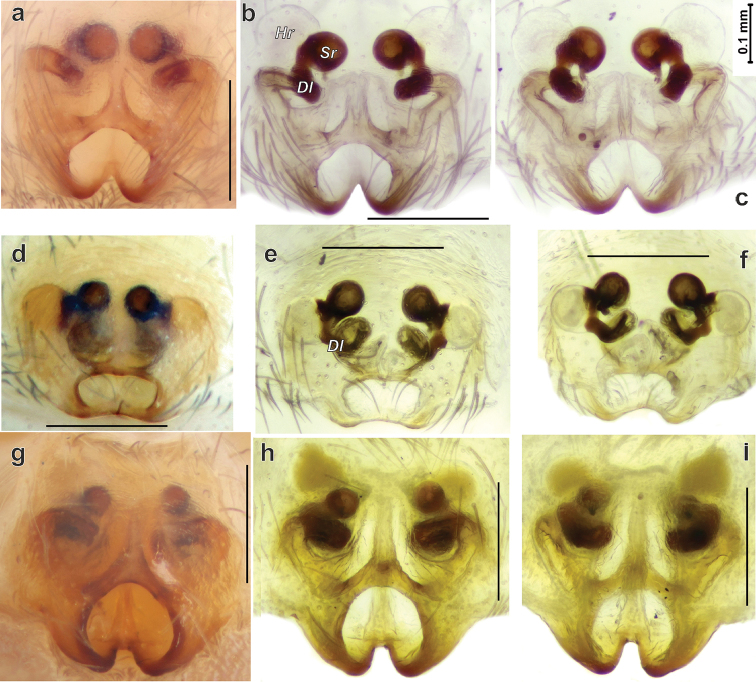
Epigyne of *Porrhoclubionamoradmandi* sp. n. (**a–c**), *P.bosmansi* sp. n. (**d–f**) and *P.leucaspis* (**g–i**). **a, d, g** intact, ventral **a, d, g** intact, ventral **b, e, h** macerated, ventral **c, f, i** macerated, dorsal. Abbreviations: *Dl*–loop of copulatory duct, *Hr*–hyaline (secondary) receptacle, *Sr*–sclerotized (primary) receptacle.

**Figure 9.**
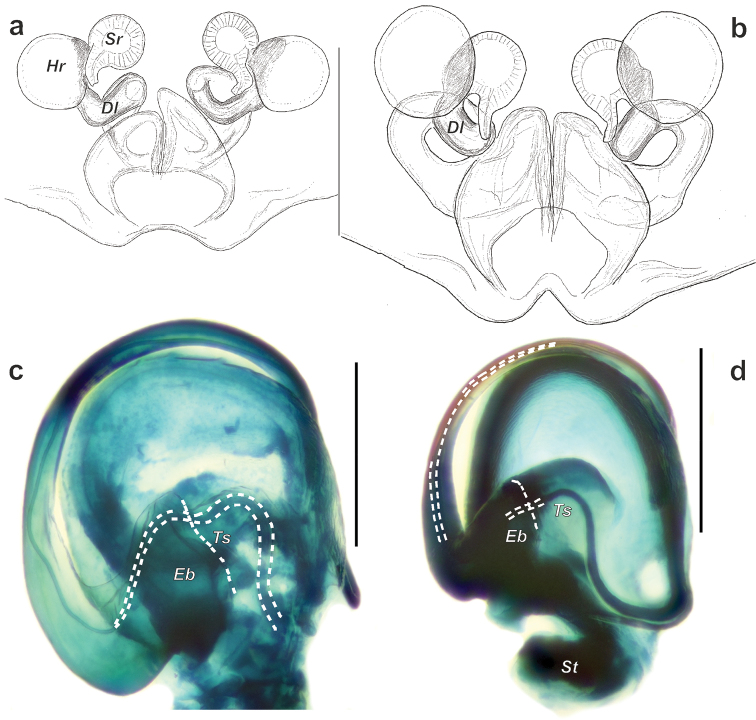
Endogyne and bulb of *Porrhoclubionabosmansi* sp. n. (**a**), *P.moradmandi* sp. n. (**b, d**) and *P.leucaspis* (**c**). **a–b** endogyne, dorsal **c–d** macerated tegulum, ventral, showing course of sperm duct. Abbreviations: *Eb*–base of embolus, *Dl*–loop of copulatory duct, *Ts*–sclerotised part of tegulum, *St*–subtegulum.

**Figure 10.**
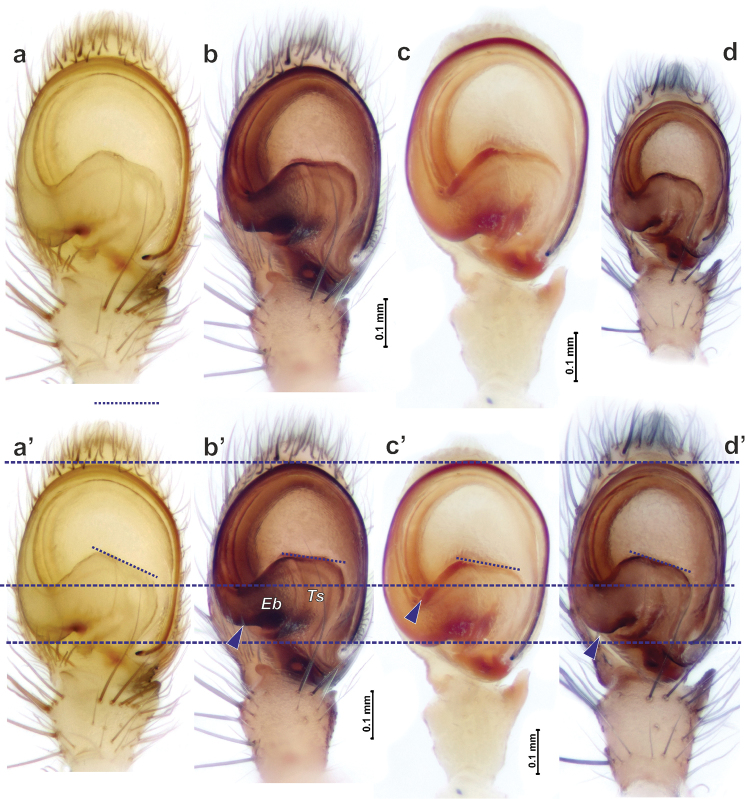
Comparison of male palp of *Porrhoclubionaleucaspis* (**a**), *P.moradmandi* sp. n. (**b**), *P.laudata* (**c**) and *P.bosmansi* sp. n. (**d**). **a–d** palps in the same scale **a’–d**’ palps shown in the same size, demonstrating different proportions. Arrows point major differenced, broken inclined line reflects differences in the angle of embolic base anterior margin, ca 25°, 8°, 10.5° and 19°. Abbreviations: *Eb*–base of embolus, *Ts*–sclerotised part of tegulum.

## Supplementary Material

XML Treatment for Clubiona

XML Treatment for Porrhoclubiona

XML Treatment for Porrhoclubiona laudata

XML Treatment for Porrhoclubiona bosmansi

XML Treatment for Porrhoclubiona moradmandi
